# Supplementary material for: A Comprehensive Analysis Examining the Role of Genetic Influences on Psychotropic Medication Response in Children
Source: Genes (Basel). 2025 Sep 8;16(9):1055. doi: 10.3390/genes16091055 (PMC12469437; doi:10.3390/genes16091055)
Supplement: Supplementary file 1 [file genes-16-01055-s001.zip › genes-3830760-supplementary.pdf]

Supplementary Table S1: Quality Appraisal using Downs and Black checklist

| ***Checklist Items*** | ***Article*** | | | | | | | | | | | | |
|---|---|---|---|---|---|---|---|---|---|---|---|---|---|
| | [7]Ramsey et al. (2020) | [22]Singh et al. (2024) | [35]Ahmed et al. (2022) | [44]Kalla et al. (2023) | [45]Concha et al. (2023) | [46]Alhazmi et al. (2022) | [47]Alyoubi et al. (2022) | [48]Firouzabadi et al. (2022) | [49]Wolking et al. (2020) | [50]Sukasem et al. (2018) | [51]Sukasem et al. (2016) | [52]Firouzabadi et al. (2016) | [53]Tsujimoto et al. (2016) |
| 1, Is the hypothesis/aim/objective of the study clearly described? (Yes: 1; No: 0) | 1 | 1 | 1 | 1 | 1 | 1 | 1 | 1 | 1 | 1 | 1 | 1 | 1 |
| 2, Are the main outcomes to be measured clearly described in the study? (Yes: 1; No: 0) | 1 | 1 | 1 | 1 | 1 | 1 | 1 | 1 | 1 | 1 | 1 | 1 | 1 |
| 3, Are the patient characteristics included in the study clearly described? (Yes: 1; No: 0) | 1 | 1 | 1 | 1 | 1 | 1 | 1 | 1 | 1 | 1 | 1 | 1 | 1 |
| 4, Are the interventions clearly described? (Yes: 1; No: 0) | 1 | 1 | 1 | 1 | 1 | 1 | 1 | 1 | 1 | 1 | 1 | 1 | 1 |
| 5, Are the distributions of principal confounders in each group of subjects clearly described? (Yes: 2; Partially: 1; No: 0) | 2 | 0 | 1 | 1 | 0 | 0 | 0 | 0 | 1 | 1 | 1 | 0 | 2 |
| 6, Are the main findings of the study clearly described? (Yes: 1; No: 0) | 1 | 1 | 1 | 1 | 1 | 1 | 1 | 1 | 1 | 1 | 1 | 1 | 1 |
| 7, Does the study estimate random variability in the data for the main outcomes? (Yes: 1; No: 0) | 1 | 1 | 1 | 1 | 1 | 1 | 1 | 1 | 1 | 1 | 1 | 1 | 1 |
| 8, Have characteristics of patients lost to follow up been described? (Yes: 1; No: 0) | 0 | N/A | 0 | 0 | 0 | 0 | 0 | 0 | 1 | 0 | 0 | 0 | 0 |

| Checklist Items | Article | | | | | | | | | | | | |
|---|---|---|---|---|---|---|---|---|---|---|---|---|---|
| | [7]Ramsey et al. (2020) | [22]Singh et al. (2024) | [35]Ahmed et al. (2022) | [44]Kalla et al. (2023) | [45]Concha et al. (2023) | [46]Alhazmi et al. (2022) | [47]Alyoubi et al. (2022) | [48]Firouzabadi et al. (2022) | [49]Wolking et al. (2020) | [50]Sukasem et al. (2018) | [51]Sukasem et al. (2016) | [52]Firouzabadi et al. (2016) | [53]Tsujimoto et al. (2016) |
| 9, Have actual probability values been reported (e.g. 0.035 rather than <0.05)? (Yes: 1; No: 0) | N/A | 1 | 1 | N/A | 1 | 1 | 1 | 1 | 1 | 1 | 1 | 1 | 1 |
| 10, Is the source of funding stated? (Yes: 1; No: 0) | 1 | 1 | 0 | 1 | 1 | 1 | 1 | 1 | 1 | 1 | 0 | N/A | 1 |
| | | | | | | | | | | | | | |
| 11, Were the subjects who were asked to participate in the study representative of the entire population? (Yes: 1; No: 0; UTD: 0) | 1 | 1 | 1 | 1 | 1 | 1 | 1 | 1 | 1 | 1 | 1 | 1 | 1 |
| 12, Were those subjects who were prepared to participate representative of the recruited population? (Yes: 1; No: 0; UTD: 0) | 1 | 1 | 1 | 1 | 1 | 1 | 1 | 1 | 1 | 1 | 1 | 1 | 1 |
| 13, Were staff, places, and facilities where patients were treated, representative of the treatment most received? (Yes: 1; No: 0; UTD: 0) | 1 | 1 | 1 | 1 | 1 | 1 | 1 | 1 | 1 | 1 | 1 | 1 | 1 |
| | | | | | | | | | | | | | |
| 14, Was an attempt made to blind study subjects to the intervention? (Yes: 1; No: 0; UTD: 0) | UTD | N/A | 0 | 0 | 0 | UTD | UTD | UTD | UTD | UTD | UTD | UTD | UTD |
| 15, Was an attempt made to blind those measuring the main outcomes? (Yes: 1; No: 0; UTD: 0) | UTD | N/A | 0 | 0 | 0 | 0 | 0 | 0 | UTD | UTD | UTD | UTD | 1 |

| ***Checklist Items*** | ***Article*** | | | | | | | | | | | | |
|---|---|---|---|---|---|---|---|---|---|---|---|---|---|
| | [7]Ramsey et al. (2020) | [22]Singh et al. (2024) | [35]Ahmed et al. (2022) | [44]Kalla et al. (2023) | [45]Concha et al. (2023) | [46]Alhazmi et al. (2022) | [47]Alyoubi et al. (2022) | [48]Firouzabadi et al. (2022) | [49]Wolking et al. (2020) | [50]Sukasem et al. (2018) | [51]Sukasem et al. (2016) | [52]Firouzabadi et al. (2016) | [53]Tsujimoto et al. (2016) |
| 16, If any of the results of the study were based on “data dredging”, was this made clear? (Yes: 1; No: 0; UTD: 0) | 1 | 1 | 1 | 1 | 1 | 1 | 1 | 1 | 1 | 1 | 1 | 1 | 1 |
| 17, Was the time between the intervention and outcome the same for cases and controls groups or adjusted for? (Yes: 1; No: 0; UTD: 0) | N/A | N/A | N/A | N/A | N/A | 1 | 1 | 1 | 1 | 1 | 1 | 1 | N/A |
| 18, Were the statistical tests used to assess the main outcomes appropriate? (Yes: 1; No: 0; UTD: 0) | 1 | 1 | 1 | 1 | 1 | 1 | 1 | 1 | 1 | 1 | 1 | 1 | 1 |
| 19, Were the main outcome measures used accurate and reliable? (Yes: 1; No: 0; UTD: 0) | 1 | 1 | 1 | 1 | 1 | 1 | 1 | 1 | 1 | 1 | 1 | 1 | 1 |
| | | | | | | | | | | | | | |
| 20, Were the patients in different intervention groups recruited from the same population? (Yes: 1; No: 0; UTD: 0) | UTD | 1 | 0 | 1 | 1 | 1 | 1 | 1 | 1 | 1 | 1 | 1 | 1 |
| 21, Were study subjects in different intervention groups (recruited over the same time period? (Yes: 1; No: 0; UTD: 0) | UTD | 1 | 1 | 1 | 0 | UTD | 0 | 1 | 1 | UTD | 1 | 1 | 1 |
| 22, Were study subjects randomised to intervention groups? (Yes: 1; No: 0; UTD: 0) | UTD | N/A | UTD | UTD | UTD | UTD | UTD | UTD | N/A | UTD | UTD | UTD | UTD |
| 23, Was the randomised intervention assignment | UTD | N/A | UTD | UTD | UTD | UTD | UTD | UTD | N/A | UTD | UTD | UTD | UTD |

| Checklist Items | Article | | | | | | | | | | | | |
|---|---|---|---|---|---|---|---|---|---|---|---|---|---|
| | [7]Ramsey et al. (2020) | [22]Singh et al. (2024) | [35]Ahmed et al. (2022) | [44]Kalla et al. (2023) | [45]Concha et al. (2023) | [46]Alhazmi et al. (2022) | [47]Alyoubi et al. (2022) | [48]Firouzabadi et al. (2022) | [49]Wolking et al. (2020) | [50]Sukasem et al. (2018) | [51]Sukasem et al. (2016) | [52]Firouzabadi et al. (2016) | [53]Tsujimoto et al. (2016) |
| concealed from both patients and staff until recruitment was completed? (Yes: 1; No: 0; UTD: 0) | | | | | | | | | | | | | |
| 24, Was there adequate adjustment for confounding in the analyses from which the main findings were drawn? (Yes: 1; No: 0; UTD: 0) | UTD | 0 | 1 | 1 | 0 | UTD | UTD | UTD | UTD | 1 | UTD | UTD | 1 |
| 25, Were losses of patients to follow-up taken into account? (Yes: 1; No: 0; UTD: 0) | 1 | N/A | UTD | UTD | UTD | UTD | UTD | UTD | 0 | UTD | UTD | UTD | UTD |
| | | | | | | | | | | | | | |
| 26, Did the study have a power calculation? Yes: 1; No: 0) | 0 | 0 | 0 | N/A | 0 | 0 | 0 | 0 | 0 | 0 | 0 | 0 | 0 |
| **Total** | 16 | 16 | 16 | 17 | 15 | 16 | 16 | 17 | 19 | 18 | 17 | 16 | 20 |

| ***Checklist Items*** | ***Article*** | | | | | | | | | | | | | | |
|---|---|---|---|---|---|---|---|---|---|---|---|---|---|---|---|
| | [54]Pouget et al. (2021) | [55]Kukec et al. (2021) | [56]Stasiołek et al. (2016) | [57]Gerlach et al. (2025) | [58]Bharthi et al. (2024) | [59]Gerlach et al. (2024) | [60]Attia et al. (2024) | [61]Gill et al. (2022) | [62]Zou et al. (2022) | [63]Nussbaum et al. (2017) | [64]Nussbaum et al. (2016) | [65]Gassó et al. (2015) | [66]Li et al. (2022) | [67]Bruxel et al. (2015) | [68]Poweleit et al. (2019) |
| | | | | | | | | | | | | | | | |
| 1, Is the hypothesis/aim/objective of the study clearly described? (Yes: 1; No: 0) | 1 | 1 | 1 | 1 | 1 | 1 | 1 | 1 | 1 | 1 | 1 | 1 | 1 | 1 | 1 |
| 2, Are the main outcomes to be measured clearly described in the study? (Yes: 1; No: 0) | 1 | 1 | 1 | 1 | 1 | 1 | 1 | 1 | 1 | 1 | 1 | 1 | 1 | 1 | 1 |
| 3, Are the patient characteristics included in the study clearly described? (Yes: 1; No: 0) | 1 | 1 | 1 | 1 | 1 | 1 | 1 | 1 | 1 | 1 | 1 | 1 | 1 | 1 | 1 |
| 4, Are the interventions clearly described? (Yes: 1; No: 0) | 1 | 1 | 1 | 1 | 1 | 1 | 1 | 1 | 1 | 1 | 1 | 1 | 1 | 1 | 1 |
| 5, Are the distributions of principal confounders in each group of subjects clearly described? (Yes: 2; Partially: 1; No: 0) | 1 | 0 | 0 | 2 | 2 | 2 | 1 | 1 | 1 | 0 | 0 | 1 | 0 | 2 | 0 |
| 6, Are the main findings of the study clearly described? (Yes: 1; No: 0) | 1 | 1 | 1 | 1 | 1 | 1 | 1 | 1 | 1 | 1 | 1 | 1 | 1 | 1 | 1 |
| 7, Does the study estimate random variability in the data for the main outcomes? (Yes: 1; No: 0) | 1 | 1 | 1 | 1 | 1 | 1 | 1 | 1 | 1 | 1 | 1 | 1 | 1 | 1 | 1 |
| 8, Have characteristics of patients lost to follow up been described? (Yes: 1; No: 0) | 1 | 1 | 0 | 0 | 0 | 0 | 0 | 0 | 1 | 0 | 0 | 0 | 1 | 1 | 0 |
| 9, Have actual probability values been reported (e.g. 0.035 | 1 | 1 | 1 | 1 | 1 | 1 | 1 | N/A | 1 | 1 | 1 | 1 | 1 | 1 | 1 |

| ***Checklist Items*** | ***Article*** | | | | | | | | | | | | | | |
|---|---|---|---|---|---|---|---|---|---|---|---|---|---|---|---|
| | [54]Pouget et al. (2021) | [55]Kukec et al. (2021) | [56]Stasiołek et al. (2016) | [57]Gerlach et al. (2025) | [58]Bharthi et al. (2024) | [59]Gerlach et al. (2024) | [60]Attia et al. (2024) | [61]Gill et al. (2022) | [62]Zou et al. (2022) | [63]Nussbaum et al. (2017) | [64]Nussbaum et al. (2016) | [65]Gassó et al. (2015) | [66]Li et al. (2022) | [67]Bruxel et al. (2015) | [68]Poweleit et al. (2019) |
| rather than <0.05)? (Yes: 1; No: 0) | | | | | | | | | | | | | | | |
| 10, Is the source of funding stated? (Yes: 1; No: 0) | 1 | 1 | 1 | 1 | 1 | 1 | 0 | 1 | 1 | 0 | 0 | 1 | 1 | 1 | 1 |
| | | | | | | | | | | | | | | | |
| 11, Were the subjects who were asked to participate in the study representative of the entire population? (Yes: 1; No: 0; UTD: 0) | 1 | 1 | 1 | 1 | 1 | 1 | 1 | 1 | 1 | 1 | 1 | 1 | 1 | 1 | 1 |
| 12, Were those subjects who were prepared to participate representative of the recruited population? (Yes: 1; No: 0; UTD: 0) | 1 | 1 | 1 | 1 | 1 | 1 | 1 | UTD | 1 | 1 | 1 | 1 | 1 | 1 | 1 |
| 13, Were staff, places, and facilities where patients were treated, representative of the treatment most received? (Yes: 1; No: 0; UTD: 0) | 1 | 1 | 1 | 1 | 1 | 1 | 1 | 1 | 1 | 1 | 1 | 1 | 1 | 1 | 1 |
| | | | | | | | | | | | | | | | |
| 14, Was an attempt made to blind study subjects to the intervention? (Yes: 1; No: 0; UTD: 0) | UTD | UTD | UTD | 0 | 0 | 0 | 1 | UTD | UTD | UTD | UTD | 0 | N/A | UTD | UTD |
| 15, Was an attempt made to blind those measuring the main outcomes? (Yes: 1; No: 0; UTD: 0) | 0 | 0 | UTD | 0 | 0 | 0 | 0 | 0 | 0 | UTD | UTD | 0 | N/A | 1 | 0 |
| 16, If any of the results of the study were based on “data dredging”, was this made clear? (Yes: 1; No: 0; UTD: 0) | 1 | 1 | 1 | 1 | 1 | 1 | 1 | 1 | 1 | 1 | 1 | 1 | 1 | 1 | 1 |

| ***Checklist Items*** | ***Article*** | | | | | | | | | | | | | | |
|---|---|---|---|---|---|---|---|---|---|---|---|---|---|---|---|
| | [54]Pouget et al. (2021) | [55]Kukec et al. (2021) | [56]Stasiołek et al. (2016) | [57]Gerlach et al. (2025) | [58]Bharthi et al. (2024) | [59]Gerlach et al. (2024) | [60]Attia et al. (2024) | [61]Gill et al. (2022) | [62]Zou et al. (2022) | [63]Nussbaum et al. (2017) | [64]Nussbaum et al. (2016) | [65]Gassó et al. (2015) | [66]Li et al. (2022) | [67]Bruxel et al. (2015) | [68]Poweleit et al. (2019) |
| 17, Was the time between the intervention and outcome the same for cases and controls groups or adjusted for? (Yes: 1; No: 0; UTD: 0) | 1 | 1 | 1 | N/A | N/A | N/A | 1 | N/A | 1 | N/A | 1 | 1 | N/A | 1 | 1 |
| 18, Were the statistical tests used to assess the main outcomes appropriate? (Yes: 1; No: 0; UTD: 0) | 1 | 1 | 1 | 1 | 1 | 1 | 1 | 1 | 1 | 1 | 1 | 1 | 1 | 1 | 1 |
| 19, Were the main outcome measures used accurate and reliable? (Yes: 1; No: 0; UTD: 0) | 1 | 1 | 1 | 1 | 1 | 1 | 1 | 1 | 1 | 1 | 1 | 1 | 1 | 1 | 1 |
| | | | | | | | | | | | | | | | |
| 20, Were the patients in different intervention groups recruited from the same population? (Yes: 1; No: 0; UTD: 0) | 1 | 1 | 1 | 1 | 0 | 0 | 0 | 1 | 1 | 1 | 1 | UTD | 1 | 1 | 1 |
| 21, Were study subjects in different intervention groups (recruited over the same time period? (Yes: 1; No: 0; UTD: 0) | 1 | 1 | 1 | 1 | UTD | UTD | 1 | UTD | UTD | 1 | 1 | UTD | UTD | UTD | 1 |
| 22, Were study subjects randomised to intervention groups? (Yes: 1; No: 0; UTD: 0) | UTD | UTD | UTD | UTD | UTD | UTD | UTD | UTD | UTD | UTD | UTD | UTD | N/A | UTD | UTD |
| 23, Was the randomised intervention assignment concealed from both patients and staff until recruitment was completed? (Yes: 1; No: 0; UTD: 0) | UTD | UTD | UTD | UTD | UTD | UTD | UTD | UTD | UTD | UTD | UTD | UTD | N/A | UTD | 1 |
| 24, Was there adequate adjustment for confounding in the analyses from which the | 1 | UTD | UTD | 1 | 1 | 1 | 1 | UTD | 1 | UTD | UTD | UTD | UTD | 1 | UTD |

| ***Checklist Items*** | ***Article*** | | | | | | | | | | | | | | |
|---|---|---|---|---|---|---|---|---|---|---|---|---|---|---|---|
| | [54]Pouget et al. (2021) | [55]Kukec et al. (2021) | [56]Stasiołek et al. (2016) | [57]Gerlach et al. (2025) | [58]Bharthi et al. (2024) | [59]Gerlach et al. (2024) | [60]Attia et al. (2024) | [61]Gill et al. (2022) | [62]Zou et al. (2022) | [63]Nussbaum et al. (2017) | [64]Nussbaum et al. (2016) | [65]Gassó et al. (2015) | [66]Li et al. (2022) | [67]Bruxel et al. (2015) | [68]Poweleit et al. (2019) |
| main findings were drawn? (Yes: 1; No: 0; UTD: 0) | | | | | | | | | | | | | | | |
| 25, Were losses of patients to follow-up taken into account? (Yes: 1; No: 0; UTD: 0) | UTD | UTD | UTD | UTD | UTD | UTD | UTD | UTD | UTD | UTD | UTD | UTD | 1 | 1 | UTD |
| | | | | | | | | | | | | | | | |
| 26, Did the study have a power calculation? Yes: 1; No: 0) | 1 | 0 | 0 | 0 | 0 | 0 | 1 | 0 | 0 | 0 | 0 | 0 | 0 | 0 | 0 |
| **Total** | 21 | 18 | 17 | 19 | 17 | 17 | 19 | 14 | 19 | 15 | 16 | 16 | 17 | 22 | 19 |

| ***Checklist Items*** | ***Article*** | | | | | | | | | | | | |
|---|---|---|---|---|---|---|---|---|---|---|---|---|---|
| | [69]Zai et al. (2023) | [70]Ivashchenko et al. (2020) | [71]Sági et al. (2021) | [72]Campagne et al. (2024) | [73]Shilbayeh et al. (2024) | [74]Honeycutt et al. (2024) | [75]Aldrich et al. (2019) | [76]Smith et al. (2017) | [77]Fan et al. (2021) | [78]Rodriguez et al. (2021) | [79]Gassó et al. (2017) | [80]Garfunkel et al. (2019) | [81]Chidambaran et al. (2015) |
| | | | | | | | | | | | | | |
| 1, Is the hypothesis/aim/objective of the study clearly described? (Yes: 1; No: 0) | 1 | 1 | 1 | 1 | 1 | 1 | 1 | 1 | 1 | 1 | 1 | 1 | 1 |
| 2, Are the main outcomes to be measured clearly described in the study? (Yes: 1; No: 0) | 1 | 1 | 1 | 1 | 1 | 1 | 1 | 1 | 1 | 1 | 1 | 1 | 1 |
| 3, Are the patient characteristics included in the study clearly described? (Yes: 1; No: 0) | 0 | 1 | 1 | 1 | 1 | 1 | 1 | 1 | 1 | 1 | 1 | 1 | 1 |
| 4, Are the interventions clearly described? (Yes: 1; No: 0) | 1 | 1 | 1 | 1 | 1 | 1 | 1 | 1 | 1 | 1 | 1 | 1 | 1 |
| 5, Are the distributions of principal confounders in each group of subjects clearly described? (Yes: 2; Partially: 1; No: 0) | 2 | 0 | 2 | 2 | 2 | 1 | 0 | 0 | 0 | 2 | 1 | 2 | 2 |
| 6, Are the main findings of the study clearly described? (Yes: 1; No: 0) | 1 | 1 | 1 | 1 | 1 | 1 | 1 | 1 | 1 | 1 | 1 | 1 | 1 |
| 7, Does the study estimate random variability in the data for the main outcomes? (Yes: 1; No: 0) | 1 | 1 | 1 | 1 | 1 | 1 | 1 | 1 | 1 | 1 | 1 | 1 | 1 |
| 8, Have characteristics of patients lost to follow up been described? (Yes: 1; No: 0) | 0 | 0 | 0 | 1 | 0 | 0 | 0 | 0 | 0 | 0 | 0 | 0 | 0 |
| 9, Have actual probability values been | 1 | 1 | 1 | 1 | 1 | 1 | 1 | 1 | N/A | 1 | 1 | 1 | 1 |

| ***Checklist Items*** | ***Article*** | | | | | | | | | | | | |
|---|---|---|---|---|---|---|---|---|---|---|---|---|---|
| | [69]Zai et al. (2023) | [70]Ivashchenko et al. (2020) | [71]Sági et al. (2021) | [72]Campagne et al. (2024) | [73]Shilbayeh et al. (2024) | [74]Honeycutt et al. (2024) | [75]Aldrich et al. (2019) | [76]Smith et al. (2017) | [77]Fan et al. (2021) | [78]Rodriguez et al. (2021) | [79]Gassó et al. (2017) | [80]Garfunkel et al. (2019) | [81]Chidambaran et al. (2015) |
| reported (e.g. 0.035 rather than <0.05)? (Yes: 1; No: 0) | | | | | | | | | | | | | |
| 10, Is the source of funding stated? (Yes: 1; No: 0) | 1 | 0 | 1 | 1 | 1 | 1 | 1 | 1 | 1 | 1 | 1 | 1 | 1 |
| | | | | | | | | | | | | | |
| 11, Were the subjects who were asked to participate in the study representative of the entire population? (Yes: 1; No: 0; UTD: 0) | UTD | 1 | 1 | 1 | 1 | 1 | 1 | 1 | 1 | 1 | 1 | 1 | 1 |
| 12, Were those subjects who were prepared to participate representative of the recruited population? (Yes: 1; No: 0; UTD: 0) | UTD | 1 | 1 | 1 | 1 | 1 | 1 | 1 | 1 | 1 | 1 | 1 | 1 |
| 13, Were staff, places, and facilities where patients were treated, representative of the treatment most received? (Yes: 1; No: 0; UTD: 0) | 1 | 1 | 1 | 1 | 1 | 1 | 1 | 1 | 1 | 1 | 1 | UTD | 1 |
| | | | | | | | | | | | | | |
| 14, Was an attempt made to blind study subjects to the intervention? (Yes: 1; No: 0; UTD: 0) | 0 | UTD | UTD | N/A | 0 | 0 | UTD | UTD | N/A | UTD | UTD | 1 | 1 |
| 15, Was an attempt made to blind those measuring the main outcomes? (Yes: 1; No: 0; UTD: 0) | 0 | UTD | UTD | N/A | 0 | 1 | 1 | UTD | N/A | UTD | UTD | 1 | 1 |
| 16, If any of the results of the study were based on | 1 | 1 | 1 | 1 | 1 | 1 | 1 | 1 | 1 | 1 | 1 | 1 | 1 |

| ***Checklist Items*** | ***Article*** | | | | | | | | | | | | |
|---|---|---|---|---|---|---|---|---|---|---|---|---|---|
| | [69]Zai et al. (2023) | [70]Ivashchenko et al. (2020) | [71]Sági et al. (2021) | [72]Campagne et al. (2024) | [73]Shilbayeh et al. (2024) | [74]Honeycutt et al. (2024) | [75]Aldrich et al. (2019) | [76]Smith et al. (2017) | [77]Fan et al. (2021) | [78]Rodriguez et al. (2021) | [79]Gassó et al. (2017) | [80]Garfunkel et al. (2019) | [81]Chidambaran et al. (2015) |
| "data dredging", was this made clear? (Yes: 1; No: 0; UTD: 0) | | | | | | | | | | | | | |
| 17, Was the time between the intervention and outcome the same for cases and controls groups or adjusted for? (Yes: 1; No: 0; UTD: 0) | N/A | 1 | 1 | N/A | UTD | 1 | 1 | 1 | N/A | 1 | N/A | 1 | N/A |
| 18, Were the statistical tests used to assess the main outcomes appropriate? (Yes: 1; No: 0; UTD: 0) | 1 | 1 | 1 | 1 | 1 | 1 | 1 | 1 | 1 | 1 | 1 | 1 | 1 |
| 19, Were the main outcome measures used accurate and reliable? (Yes: 1; No: 0; UTD: 0) | 1 | 1 | 1 | 1 | 1 | 1 | 1 | 1 | 1 | 1 | 1 | 1 | 1 |
| | | | | | | | | | | | | | |
| 20, Were the patients in different intervention groups recruited from the same population? (Yes: 1; No: 0; UTD: 0) | UTD | 1 | 0 | 1 | 0 | 1 | 1 | 1 | 1 | 1 | 1 | 0 | 1 |
| 21, Were study subjects in different intervention groups (recruited over the same time period? (Yes: 1; No: 0; UTD: 0) | 0 | 1 | UTD | 1 | 1 | UTD | 1 | 1 | 1 | UTD | UTD | UTD | 1 |
| 22, Were study subjects randomised to intervention groups? (Yes: 1; No: 0; UTD: 0) | UTD | UTD | UTD | 1 | UTD | N/A | UTD | UTD | N/A | UTD | UTD | 1 | UTD |
| 23, Was the randomised intervention assignment concealed from both patients and staff until recruitment was completed? (Yes: 1; No: 0; UTD: 0) | UTD | UTD | UTD | UTD | UTD | N/A | UTD | UTD | N/A | UTD | UTD | 1 | UTD |

| ***Checklist Items*** | ***Article*** | | | | | | | | | | | | |
|---|---|---|---|---|---|---|---|---|---|---|---|---|---|
| | [69]Zai et al. (2023) | [70]Ivashchenko et al. (2020) | [71]Sági et al. (2021) | [72]Campagne et al. (2024) | [73]Shilbayeh et al. (2024) | [74]Honeycutt et al. (2024) | [75]Aldrich et al. (2019) | [76]Smith et al. (2017) | [77]Fan et al. (2021) | [78]Rodriguez et al. (2021) | [79]Gassó et al. (2017) | [80]Garfunkel et al. (2019) | [81]Chidambaran et al. (2015) |
| 24, Was there adequate adjustment for confounding in the analyses from which the main findings were drawn? (Yes: 1; No: 0; UTD: 0) | 1 | UTD | 1 | 1 | 1 | 1 | UTD | UTD | UTD | 1 | 1 | 1 | UTD |
| 25, Were losses of patients to follow-up taken into account? (Yes: 1; No: 0; UTD: 0) | UTD | UTD | UTD | UTD | UTD | UTD | UTD | UTD | UTD | UTD | UTD | UTD | UTD |
| | | | | | | | | | | | | | |
| 26, Did the study have a power calculation? Yes: 1; No: 0) | 0 | 0 | 1 | 0 | 0 | 0 | 0 | 0 | 0 | 0 | 1 | 1[Ψ] | 1 |
| **Total** | 14 | 16 | 19 | 21 | 18 | 19 | 18 | 17 | 15 | 19 | 18 | 22 | 21 |

[Ψ]Although a power calculation could not be identified, the secondary analysis that investigated three-way treatment interaction did reduce the statistical power by half.

| Checklist Items | Article | | | | | | | | |
|---|---|---|---|---|---|---|---|---|---|
| | [82]Sadhasivam et al. (2015) | [83]Vande Voort et al. (2022) | [84]Nooraeen et al. (2024) | [85]Liko et al. (2021) | [86]Cohn et al. (2021) | [87]Davis et al. (2021) | [88]Gota et al. (2016) | [89]Hallik et al. (2022) | [90]Johnson et al. (2021) |
| | | | | | | | | | |
| 1, Is the hypothesis/aim/objective of the study clearly described? (Yes: 1; No: 0) | 1 | 1 | 1 | 1 | 1 | 1 | 1 | 1 | 1 |
| 2, Are the main outcomes to be measured clearly described in the study? (Yes: 1; No: 0) | 1 | 1 | 1 | 1 | 1 | 1 | 1 | 1 | 1 |
| 3, Are the patient characteristics included in the study clearly described? (Yes: 1; No: 0) | 1 | 1 | 1 | 1 | 1 | 1 | 1 | 1 | 1 |
| 4, Are the interventions clearly described? (Yes: 1; No: 0) | 1 | 1 | 1 | 1 | 1 | 1 | 1 | 1 | 1 |
| 5, Are the distributions of principal confounders in each group of subjects clearly described? (Yes: 2; Partially: 1; No: 0) | 1 | 0 | 0 | 1 | 0 | 2 | 0 | 0 | 0 |
| 6, Are the main findings of the study clearly described? (Yes: 1; No: 0) | 1 | 1 | 1 | 1 | 1 | 1 | 1 | 1 | 1 |
| 7, Does the study estimate random variability in the data for the main outcomes? (Yes: 1; No: 0) | 1 | 1 | 1 | 1 | 1 | 1 | 1 | 1 | 1 |
| 8, Have characteristics of patients lost to follow up been described? (Yes: 1; No: 0) | 0 | 1 | 0 | N/A | 0 | 0 | 0 | 0 | 0 |
| 9, Have actual probability values been reported (e.g. 0.035 rather than <0.05)? (Yes: 1; No: 0) | 1 | 1 | 1 | 1 | 0 | 1 | 0 | 1 | 1 |
| 10, Is the source of funding stated? (Yes: 1; No: 0) | 1 | 1 | 1 | 1 | 1 | 1 | 1 | 1 | 1 |
| | | | | | | | | | |
| 11, Were the subjects who were asked to participate in the study representative of the entire population? (Yes: 1; No: 0; UTD: 0) | 1 | 1 | 1 | 1 | 1 | 1 | 1 | 1 | 1 |
| 12, Were those subjects who were prepared to participate representative of the recruited population? (Yes: 1; No: 0; UTD: 0) | 1 | 1 | 1 | 1 | 1 | 1 | 1 | 1 | 1 |
| 13, Were staff, places, and facilities where patients were treated, representative of the treatment most received? (Yes: 1; No: 0; UTD: 0) | 1 | 1 | 1 | 1 | 1 | 1 | 1 | 1 | 1 |
| | | | | | | | | | |
| 14, Was an attempt made to blind study subjects to the intervention? (Yes: 1; No: 0; UTD: 0) | 1 | 1 | N/A | N/A | UTD | UTD | UTD | N/A | UTD |
| 15, Was an attempt made to blind those measuring the main outcomes? (Yes: 1; No: 0; UTD: 0) | 1 | 1 | N/A | N/A | 0 | 0 | UTD | N/A | 0 |

| *Checklist Items* | *Article* | | | | | | | | |
|---|---|---|---|---|---|---|---|---|---|
| | [82]Sadhasivam et al. (2015) | [83]Vande Voort et al. (2022) | [84]Nooraeen et al. (2024) | [85]Liko et al. (2021) | [86]Cohn et al. (2021) | [87]Davis et al. (2021) | [88]Gota et al. (2016) | [89]Hallik et al. (2022) | [90]Johnson et al. (2021) |
| 16, If any of the results of the study were based on "data dredging", was this made clear? (Yes: 1; No: 0; UTD: 0) | 1 | 1 | 1 | 1 | 1 | 1 | 1 | 1 | 1 |
| 17, Was the time between the intervention and outcome the same for cases and controls groups or adjusted for? (Yes: 1; No: 0; UTD: 0) | N/A | 1 | N/A | N/A | N/A | N/A | N/A | N/A | N/A |
| 18, Were the statistical tests used to assess the main outcomes appropriate? (Yes: 1; No: 0; UTD: 0) | 1 | 1 | 1 | 1 | 1 | 1 | 1 | 1 | 1 |
| 19, Were the main outcome measures used accurate and reliable? (Yes: 1; No: 0; UTD: 0) | 1 | 1 | 1 | 1 | 1 | 1 | 1 | 1 | 1 |
| | | | | | | | | | |
| 20, Were the patients in different intervention groups recruited from the same population? (Yes: 1; No: 0; UTD: 0) | 1 | 1 | 1 | 1 | 1 | 1 | 1 | 1 | 1 |
| 21, Were study subjects in different intervention groups (recruited over the same time period? (Yes: 1; No: 0; UTD: 0) | 1 | 1 | 1 | 1 | 1 | 1 | 1 | 1 | 1 |
| 22, Were study subjects randomised to intervention groups? (Yes: 1; No: 0; UTD: 0) | UTD | 1 | 1 | UTD | UTD | UTD | UTD | N/A | UTD |
| 23, Was the randomised intervention assignment concealed from both patients and staff until recruitment was completed? (Yes: 1; No: 0; UTD: 0) | UTD | 1 | 1 | UTD | UTD | UTD | UTD | N/A | UTD |
| 24, Was there adequate adjustment for confounding in the analyses from which the main findings were drawn? (Yes: 1; No: 0; UTD: 0) | 1 | UTD | UTD | 1 | UTD | 1 | 1 | UTD | UTD |
| 25, Were losses of patients to follow-up taken into account? (Yes: 1; No: 0; UTD: 0) | UTD | 1 | UTD | N/A | UTD | UTD | UTD | UTD | UTD |
| | | | | | | | | | |
| 26, Did the study have a power calculation? Yes: 1; No: 0) | 1 | 1 | 0 | 0 | 0 | 0 | 0 | 0 | 0 |
| **Total** | 21 | 24 | 18 | 18 | 15 | 19 | 16 | 16 | 16 |

Abbreviations: N/A (Not Applicable); UTD (Unable to determine)

Notes:
From Downs and Black (1998) checklist [41].

Not applicable denotes when a checklist item is not relevant.

Supplementary Table S2: Health Economic Evaluation using the Consolidated Health Economic Evaluation Reporting Standards (CHEERS) checklist

| ***Checklist Items*** | ***Article*** | | | | | | | | | | | | |
|---|---|---|---|---|---|---|---|---|---|---|---|---|---|
| | [7]Ramsey et al. (2020) | [22]Singh et al. (2024) | [35]Ahmed et al. (2022) | [44]Kalla et al. (2023) | [45]Concha et al. (2023) | [46]Alhazmi et al. (2022) | [47]Alyoubi et al. (2022) | [48]Firouzabadi et al. (2022) | [49]Wolking et al. (2020) | [50]Sukasem et al. (2018) | [51]Sukasem et al. (2016) | [52]Firouzabadi et al. (2016) | [53]Tsujimoto et al. (2016) |
| 1, Title | 0 | 0 | 0 | 0 | 0 | 0 | 0 | 0 | 0 | 0 | 0 | 0 | 0 |
| 2, Abstract | 1 | 1 | 1 | 1 | 1 | 1 | 1 | 1 | 1 | 1 | 1 | 1 | 1 |
| 3, Background and objectives | 1 | 1 | 1 | 1 | 1 | 1 | 1 | 1 | 1 | 1 | 1 | 1 | 1 |
| 4, Health economic analysis plan | 0 | 0 | 0 | 0 | 0 | 0 | 0 | 0 | 0 | 0 | 0 | 0 | 0 |
| 5, Study population | 1 | 1 | 1 | 1 | 1 | 1 | 1 | 1 | 1 | 1 | 1 | 1 | 1 |
| 6, Setting and location | 1 | 1 | 1 | 1 | 1 | 1 | 1 | 1 | 1 | 1 | 1 | 1 | 1 |
| 7, Comparators | N/A | N/A | N/A | N/A | N/A | N/A | N/A | N/A | N/A | N/A | N/A | N/A | N/A |
| 8, Perspective | N/A | N/A | N/A | N/A | N/A | N/A | N/A | N/A | N/A | N/A | N/A | N/A | N/A |
| 9, Time horizon | N/A | N/A | N/A | N/A | N/A | N/A | N/A | N/A | N/A | N/A | N/A | N/A | N/A |
| 10, Discount rate | N/A | N/A | N/A | N/A | N/A | N/A | N/A | N/A | N/A | N/A | N/A | N/A | N/A |
| 11, Selection of outcomes | N/A | N/A | N/A | N/A | N/A | N/A | N/A | N/A | N/A | N/A | N/A | N/A | N/A |
| 12, Measurement of outcomes | N/A | N/A | N/A | N/A | N/A | N/A | N/A | N/A | N/A | N/A | N/A | N/A | N/A |
| 13, Valuation of outcomes | N/A | N/A | N/A | N/A | N/A | N/A | N/A | N/A | N/A | N/A | N/A | N/A | N/A |
| 14, Measurement and valuation of resources and costs | N/A | N/A | N/A | N/A | N/A | N/A | N/A | N/A | N/A | N/A | N/A | N/A | N/A |
| 15, Currency, price date, and conversion | N/A | N/A | N/A | N/A | N/A | N/A | N/A | N/A | N/A | N/A | N/A | N/A | N/A |

| Checklist Items | Article | | | | | | | | | | | | |
|---|---|---|---|---|---|---|---|---|---|---|---|---|---|
| | [7]Ramsey et al. (2020) | [22]Singh et al. (2024) | [35]Ahmed et al. (2022) | [44]Kalla et al. (2023) | [45]Concha et al. (2023) | [46]Alhazmi et al. (2022) | [47]Alyoubi et al. (2022) | [48]Firouzabadi et al. (2022) | [49]Wolking et al. (2020) | [50]Sukasem et al. (2018) | [51]Sukasem et al. (2016) | [52]Firouzabadi et al. (2016) | [53]Tsujimoto et al. (2016) |
| 16, Rationale and description of model | 0 | 0 | 0 | 0 | 0 | 0 | 0 | 0 | 0 | 0 | 0 | 0 | 0 |
| 17, Analytics and assumptions | 0 | 0 | 0 | 0 | 0 | 0 | 0 | 0 | 0 | 0 | 0 | 0 | 0 |
| 18, Characterizing heterogeneity | 1 | 0 | 1 | 1 | 0 | 0 | 0 | 0 | 1 | 1 | 1 | 0 | 1 |
| 19, Characterising distributional effects | 0 | 0 | 0 | 0 | 0 | 0 | 0 | 0 | 0 | 0 | 0 | 0 | 0 |
| 20, Characterizing uncertainty | 0 | 0 | 0 | 0 | 0 | 0 | 0 | 0 | 0 | 0 | 0 | 0 | 0 |
| 21, Approach to engagement with patients and others affected by the study | 0 | 0 | 0 | 0 | 0 | 0 | 0 | 0 | 0 | 0 | 0 | 0 | 0 |
| 22, Study parameters | 1 | 1 | 1 | 1 | 1 | 1 | 1 | 1 | 1 | 1 | 1 | 1 | 1 |
| 23, Summary of main results | 1 | 1 | 1 | 1 | 1 | 1 | 1 | 1 | 1 | 1 | 1 | 1 | 1 |
| 24, Effect of uncertainty | 0 | 0 | 0 | 0 | 0 | 0 | 0 | 0 | 0 | 0 | 0 | 0 | 0 |
| 25, Effect of engagement with patients and others affected by the study | 0 | 0 | 0 | 0 | 0 | 0 | 0 | 0 | 0 | 0 | 0 | 0 | 0 |
| 26, Study findings, limitations, | 1 | 1 | 1 | 1 | 1 | 0 | 1 | 1 | 1 | 1 | 1 | 0 | 1 |

| ***Checklist Items*** | ***Article*** | | | | | | | | | | | | |
|---|---|---|---|---|---|---|---|---|---|---|---|---|---|
| | [7]Ramsey et al. (2020) | [22]Singh et al. (2024) | [35]Ahmed et al. (2022) | [44]Kalla et al. (2023) | [45]Concha et al. (2023) | [46]Alhazmi et al. (2022) | [47]Alyoubi et al. (2022) | [48]Firouzabadi et al. (2022) | [49]Wolking et al. (2020) | [50]Sukasem et al. (2018) | [51]Sukasem et al. (2016) | [52]Firouzabadi et al. (2016) | [53]Tsujimoto et al. (2016) |
| generalizability, and current knowledge | | | | | | | | | | | | | |
| 27, Source of funding | 1 | 1 | 0 | 1 | 1 | 1 | 1 | 0 | 1 | 1 | 0 | 1 | 1 |
| 28, Conflicts of interest | 1 | 1 | 1 | 1 | 1 | 1 | 1 | 1 | 1 | 0 | 1 | 1 | 1 |
| **Total** | 10 | 9 | 9 | 10 | 9 | 8 | 9 | 8 | 10 | 9 | 9 | 8 | 10 |
| **% Reported (excl. N/A)** | 35.71% | 32.14% | 32.14% | 35.71% | 32.14% | 28.57% | 32.14% | 28.57% | 35.71% | 32.14% | 32.14% | 28.57% | 35.71% |

| *Checklist Items* | *Article* | | | | | | | | | | | | | | |
|---|---|---|---|---|---|---|---|---|---|---|---|---|---|---|---|
| | [54]Pouget et al. (2021) | [55]Kukec et al. (2021) | [56]Stasiołek et al. (2016) | [57]Gerlach et al. (2025) | [58]Bharthi et al. (2024) | [59]Gerlach et al. (2024) | [60]Attia et al. (2024) | [61]Gill et al. (2022) | [62]Zou et al. (2022) | [63]Nussbaum et al. (2017) | [64]Nussbaum et al. (2016) | [65]Gassó et al. (2015) | [66]Li et al. (2022) | [67]Bruxel et al. (2015) | [68]Poweleit et al. (2019) |
| 1, Title | 0 | 0 | 0 | 0 | 0 | 0 | 0 | 0 | 0 | 0 | 0 | 0 | 0 | 0 | 0 |
| 2, Abstract | 1 | 1 | 1 | 1 | 1 | 1 | 1 | 1 | 1 | 1 | 1 | 1 | 1 | 1 | 1 |
| 3, Background and objectives | 1 | 1 | 1 | 1 | 1 | 1 | 1 | 1 | 1 | 1 | 1 | 1 | 1 | 1 | 1 |
| 4, Health economic analysis plan | 0 | 0 | 0 | 0 | 0 | 0 | 0 | 0 | 0 | 0 | 0 | 0 | 0 | 0 | 0 |
| 5, Study population | 1 | 1 | 1 | 1 | 1 | 1 | 1 | 1 | 1 | 1 | 1 | 1 | 1 | 1 | 1 |
| 6, Setting and location | 1 | 1 | 1 | 1 | 1 | 1 | 1 | 1 | 1 | 1 | 1 | 1 | 1 | 1 | 1 |
| 7, Comparators | N/A | N/A | N/A | N/A | N/A | N/A | N/A | N/A | N/A | N/A | N/A | N/A | N/A | N/A | N/A |
| 8, Perspective | N/A | N/A | N/A | N/A | N/A | N/A | N/A | N/A | N/A | N/A | N/A | N/A | N/A | N/A | N/A |
| 9, Time horizon | N/A | N/A | N/A | N/A | N/A | N/A | N/A | N/A | N/A | N/A | N/A | N/A | N/A | N/A | N/A |
| 10, Discount rate | N/A | N/A | N/A | N/A | N/A | N/A | N/A | N/A | N/A | N/A | N/A | N/A | N/A | N/A | N/A |
| 11, Selection of outcomes | N/A | N/A | N/A | N/A | N/A | N/A | N/A | N/A | N/A | N/A | N/A | N/A | N/A | N/A | N/A |
| 12, Measurement of outcomes | N/A | N/A | N/A | N/A | N/A | N/A | N/A | N/A | N/A | N/A | N/A | N/A | N/A | N/A | N/A |
| 13, Valuation of outcomes | N/A | N/A | N/A | N/A | N/A | N/A | N/A | N/A | N/A | N/A | N/A | N/A | N/A | N/A | N/A |
| 14, Measurement and valuation of resources and costs | N/A | N/A | N/A | N/A | N/A | N/A | N/A | N/A | N/A | N/A | N/A | N/A | N/A | N/A | N/A |
| 15, Currency, price date, and conversion | N/A | N/A | N/A | N/A | N/A | N/A | N/A | N/A | N/A | N/A | N/A | N/A | N/A | N/A | N/A |
| 16, Rationale and description of model | 0 | 0 | 0 | 0 | 0 | 0 | 0 | 0 | 0 | 0 | 0 | 0 | 0 | 0 | 0 |

| ***Checklist Items*** | ***Article*** | | | | | | | | | | | | | | |
|---|---|---|---|---|---|---|---|---|---|---|---|---|---|---|---|
| | [54]Pouget et al. (2021) | [55]Kukec et al. (2021) | [56]Stasiołek et al. (2016) | [57]Gerlach et al. (2025) | [58]Bharthi et al. (2024) | [59]Gerlach et al. (2024) | [60]Attia et al. (2024) | [61]Gill et al. (2022) | [62]Zou et al. (2022) | [63]Nussbaum et al. (2017) | [64]Nussbaum et al. (2016) | [65]Gassó et al. (2015) | [66]Li et al. (2022) | [67]Bruxel et al. (2015) | [68]Poweleit et al. (2019) |
| 17, Analytics and assumptions | 0 | 0 | 0 | 0 | 0 | 0 | 0 | 0 | 0 | 0 | 0 | 0 | 0 | 0 | 0 |
| 18, Characterizing heterogeneity | 1 | 0 | 0 | 1 | 1 | 1 | 1 | 0 | 0 | 0 | 0 | 1 | 0 | 1 | 0 |
| 19, Characterising distributional effects | 0 | 0 | 0 | 0 | 0 | 0 | 0 | 0 | 0 | 0 | 0 | 0 | 0 | 0 | 0 |
| 20, Characterizing uncertainty | 0 | 0 | 0 | 0 | 0 | 0 | 0 | 0 | 0 | 0 | 0 | 0 | 0 | 0 | 0 |
| 21, Approach to engagement with patients and others affected by the study | 0 | 0 | 0 | 0 | 0 | 0 | 0 | 0 | 0 | 0 | 0 | 0 | 0 | 0 | 0 |
| 22, Study parameters | 1 | 1 | 1 | 1 | 1 | 1 | 1 | 1 | 1 | 1 | 1 | 1 | 1 | 1 | 1 |
| 23, Summary of main results | 1 | 1 | 1 | 1 | 1 | 1 | 1 | 1 | 1 | 1 | 1 | 1 | 1 | 1 | 1 |
| 24, Effect of uncertainty | 0 | 0 | 0 | 0 | 0 | 0 | 0 | 0 | 0 | 0 | 0 | 0 | 0 | 0 | 0 |
| 25, Effect of engagement with patients and others affected by the study | 0 | 0 | 0 | 0 | 0 | 0 | 0 | 0 | 0 | 0 | 0 | 0 | 0 | 0 | 0 |
| 26, Study findings, limitations, generalizability, and | 1 | 1 | 1 | 1 | 1 | 1 | 1 | 0 | 1 | 0 | 0 | 1 | 1 | 1 | 1 |

| *Checklist Items* | *Article* | | | | | | | | | | | | | | |
|---|---|---|---|---|---|---|---|---|---|---|---|---|---|---|---|
| | [54]Pouget et al. (2021) | [55]Kukec et al. (2021) | [56]Stasiołek et al. (2016) | [57]Gerlach et al. (2025) | [58]Bharthi et al. (2024) | [59]Gerlach et al. (2024) | [60]Attia et al. (2024) | [61]Gill et al. (2022) | [62]Zou et al. (2022) | [63]Nussbaum et al. (2017) | [64]Nussbaum et al. (2016) | [65]Gassó et al. (2015) | [66]Li et al. (2022) | [67]Bruxel et al. (2015) | [68]Poweleit et al. (2019) |
| current knowledge | | | | | | | | | | | | | | | |
| 27, Source of funding | 1 | 1 | 1 | 1 | 1 | 1 | 0[Ψ] | 1 | 1 | 0 | 0 | 1 | 1 | 1 | 1 |
| 28, Conflicts of interest | 1 | 1 | 1 | 1 | 1 | 1 | 1 | 1 | 1 | 0 | 1 | 1 | 1 | 1 | 1 |
| **Total** | 10 | 9 | 9 | 10 | 10 | 10 | 9 | 8 | 9 | 6 | 7 | 10 | 9 | 10 | 9 |
| **% Reported (excl. N/A)** | 35.71% | 32.14% | 32.14% | 35.71% | 35.71% | 35.71% | 32.14% | 28.57% | 32.14% | 21.43% | 25.00% | 35.71% | 32.14% | 35.71% | 32.14% |

[Ψ]Funding acquisition was mentioned but no details of funding was provided.

| ***Checklist Items*** | ***Article*** | | | | | | | | | | | | |
|---|---|---|---|---|---|---|---|---|---|---|---|---|---|
| | [69]Zai et al. (2023) | [70]Ivashchenko et al. (2020) | [71]Sági et al. (2021) | [72]Campagne et al. (2024) | [73]Shilbayeh et al. (2024) | [74]Honeycutt et al. (2024) | [75]Aldrich et al. (2019) | [76]Smith et al. (2017) | [77]Fan et al. (2021) | [78]Rodriguez et al. (2021) | [79]Gassó et al. (2017) | [80]Garfunkel et al. (2019) | [81]Chidambaran et al. (2015) |
| 1, Title | 0 | 0 | 0 | 0 | 0 | 0 | 0 | 0 | 0 | 0 | 0 | 0 | 0 |
| 2, Abstract | 1 | 1 | 1 | 1 | 1 | 1 | 1 | 1 | 1 | 1 | 1 | 1 | 1 |
| 3, Background and objectives | 1 | 1 | 1 | 1 | 1 | 1 | 1 | 1 | 1 | 1 | 1 | 1 | 1 |
| 4, Health economic analysis plan | 0 | 0 | 0 | 0 | 0 | 0 | 0 | 0 | 0 | 0 | 0 | 0 | 0 |
| 5, Study population | 1 | 1 | 1 | 1 | 1 | 1 | 1 | 1 | 1 | 1 | 1 | 1 | 1 |
| 6, Setting and location | 1 | 1 | 1 | 1 | 1 | 1 | 1 | 1 | 1 | 1 | 1 | 1 | 1 |
| 7, Comparators | N/A | N/A | N/A | N/A | N/A | N/A | N/A | N/A | N/A | N/A | N/A | N/A | N/A |
| 8, Perspective | N/A | N/A | N/A | N/A | N/A | N/A | N/A | N/A | N/A | N/A | N/A | N/A | N/A |
| 9, Time horizon | N/A | N/A | N/A | N/A | N/A | N/A | N/A | N/A | N/A | N/A | N/A | N/A | N/A |
| 10, Discount rate | N/A | N/A | N/A | N/A | N/A | N/A | N/A | N/A | N/A | N/A | N/A | N/A | N/A |
| 11, Selection of outcomes | N/A | N/A | N/A | N/A | N/A | N/A | N/A | N/A | N/A | N/A | N/A | N/A | N/A |
| 12, Measurement of outcomes | N/A | N/A | N/A | N/A | N/A | N/A | N/A | N/A | N/A | N/A | N/A | N/A | N/A |
| 13, Valuation of outcomes | N/A | N/A | N/A | N/A | N/A | N/A | N/A | N/A | N/A | N/A | N/A | N/A | N/A |
| 14, Measurement and valuation of resources and costs | N/A | N/A | N/A | N/A | N/A | N/A | N/A | N/A | N/A | N/A | N/A | N/A | N/A |
| 15, Currency, price date, and conversion | N/A | N/A | N/A | N/A | N/A | N/A | N/A | N/A | N/A | N/A | N/A | N/A | N/A |
| 16, Rationale and description of model | 0 | 0 | 0 | 0 | 0 | 0 | 0 | 0 | 0 | 0 | 0 | 0 | 0 |

| Checklist Items | Article | | | | | | | | | | | | |
|---|---|---|---|---|---|---|---|---|---|---|---|---|---|
| | [69]Zai et al. (2023) | [70]Ivashchenko et al. (2020) | [71]Sági et al. (2021) | [72]Campagne et al. (2024) | [73]Shilbayeh et al. (2024) | [74]Honeycutt et al. (2024) | [75]Aldrich et al. (2019) | [76]Smith et al. (2017) | [77]Fan et al. (2021) | [78]Rodriguez et al. (2021) | [79]Gassó et al. (2017) | [80]Garfunkel et al. (2019) | [81]Chidambaran et al. (2015) |
| 17, Analytics and assumptions | 0 | 0 | 0 | 0 | 0 | 0 | 0 | 0 | 0 | 0 | 0 | 0 | 0 |
| 18, Characterizing heterogeneity | 1 | 0 | 1 | 1 | 1 | 1 | 0 | 0 | 1 | 1 | 1 | 1 | 1 |
| 19, Characterising distributional effects | 0 | 0 | 0 | 0 | 0 | 0 | 0 | 0 | 0 | 0 | 0 | 0 | 0 |
| 20, Characterizing uncertainty | 0 | 0 | 0 | 0 | 0 | 0 | 0 | 0 | 0 | 0 | 0 | 0 | 0 |
| 21, Approach to engagement with patients and others affected by the study | 0 | 0 | 0 | 0 | 0 | 0 | 0 | 0 | 0 | 0 | 0 | 0 | 0 |
| 22, Study parameters | 1 | 1 | 1 | 1 | 1 | 1 | 1 | 1 | 1 | 1 | 1 | 1 | 1 |
| 23, Summary of main results | 1 | 1 | 1 | 1 | 1 | 1 | 1 | 1 | 1 | 1 | 1 | 1 | 1 |
| 24, Effect of uncertainty | 0 | 0 | 0 | 0 | 0 | 0 | 0 | 0 | 0 | 0 | 0 | 0 | 0 |
| 25, Effect of engagement with patients and others affected by the study | 0 | 0 | 0 | 0 | 0 | 0 | 0 | 0 | 0 | 0 | 0 | 0 | 0 |
| 26, Study findings, limitations, generalizability, and current knowledge | 1 | 1 | 1 | 1 | 1 | 1 | 1 | 1 | 1 | 1 | 1 | 1 | 1 |

| *Checklist Items* | *Article* | | | | | | | | | | | | |
|---|---|---|---|---|---|---|---|---|---|---|---|---|---|
| | [69]Zai et al. (2023) | [70]Ivashchenko et al. (2020) | [71]Sági et al. (2021) | [72]Campagne et al. (2024) | [73]Shilbayeh et al. (2024) | [74]Honeycutt et al. (2024) | [75]Aldrich et al. (2019) | [76]Smith et al. (2017) | [77]Fan et al. (2021) | [78]Rodriguez et al. (2021) | [79]Gassó et al. (2017) | [80]Garfunkel et al. (2019) | [81]Chidambaran et al. (2015) |
| 27, Source of funding | 1 | 0 | 1 | 1 | 1 | 1 | 1 | 1 | 1 | 1 | 1 | 1 | 1 |
| 28, Conflicts of interest | 1 | 1 | 1 | 1 | 1 | 1 | 1 | 1 | 1 | 1 | 0 | 1 | 1 |
| **Total** | 10 | 8 | 10 | 10 | 10 | 10 | 9 | 9 | 10 | 10 | 9 | 10 | 10 |
| **% Reported (excl. N/A)** | 35.71% | 28.57% | 35.71% | 35.71% | 35.71% | 35.71% | 32.14% | 32.14% | 35.71% | 35.71% | 32.14% | 35.71% | 35.71% |

| Checklist Items | *Article* | | | | | | | | |
|---|---|---|---|---|---|---|---|---|---|
| | [82]Sadhasivam et al. (2015) | [83]Vande Voort et al. (2022) | [84]Nooraeen et al. (2024) | [85]Liko et al. (2021) | [86]Cohn et al. (2021) | [87]Davis et al. (2021) | [88]Gota et al. (2016) | [89]Hallik et al. (2022) | [90]Johnson et al. (2021) |
| 1, Title | 0 | 0 | 0 | 0 | 0 | 0 | 0 | 0 | 0 |
| 2, Abstract | 1 | 1 | 1 | 1 | 1 | 1 | 1 | 1 | 1 |
| 3, Background and objectives | 1 | 1 | 1 | 1 | 1 | 1 | 1 | 1 | 1 |
| 4, Health economic analysis plan | 0 | 0 | 0 | 0 | 0 | 0 | 0 | 0 | 0 |
| 5, Study population | 1 | 1 | 1 | 1 | 1 | 1 | 1 | 1 | 1 |
| 6, Setting and location | 1 | 1 | 1 | 1 | 1 | 1 | 1 | 1 | 1 |
| 7, Comparators | N/A | N/A | N/A | N/A | N/A | N/A | N/A | N/A | N/A |
| 8, Perspective | N/A | N/A | N/A | N/A | N/A | N/A | N/A | N/A | N/A |
| 9, Time horizon | N/A | N/A | N/A | N/A | N/A | N/A | N/A | N/A | N/A |
| 10, Discount rate | N/A | N/A | N/A | N/A | N/A | N/A | N/A | N/A | N/A |
| 11, Selection of outcomes | N/A | N/A | N/A | N/A | N/A | N/A | N/A | N/A | N/A |
| 12, Measurement of outcomes | N/A | N/A | N/A | N/A | N/A | N/A | N/A | N/A | N/A |
| 13, Valuation of outcomes | N/A | N/A | N/A | N/A | N/A | N/A | N/A | N/A | N/A |
| 14, Measurement and valuation of resources and costs | N/A | N/A | N/A | N/A | N/A | N/A | N/A | N/A | N/A |
| 15, Currency, price date, and conversion | N/A | N/A | N/A | N/A | N/A | N/A | N/A | N/A | N/A |
| 16, Rationale and description of model | 0 | 0 | 0 | 0 | 0 | 0 | 0 | 0 | 0 |
| 17, Analytics and assumptions | 0 | 0 | 0 | 0 | 0 | 0 | 0 | 0 | 0 |
| 18, Characterizing heterogeneity | 1 | 0 | 0 | 1 | 1 | 0 | 0 | 1 | 0 |
| 19, Characterising distributional effects | 0 | 0 | 0 | 0 | 0 | 0 | 0 | 0 | 0 |
| 20, Characterizing uncertainty | 0 | 0 | 0 | 0 | 0 | 0 | 0 | 0 | 0 |
| 21, Approach to engagement with patients and others affected by the study | 0 | 0 | 0 | 0 | 0 | 0 | 0 | 0 | 0 |
| 22, Study parameters | 1 | 1 | 1 | 1 | 1 | 1 | 1 | 1 | 1 |
| 23, Summary of main results | 1 | 1 | 1 | 1 | 1 | 1 | 1 | 1 | 1 |
| 24, Effect of uncertainty | 0 | 0 | 0 | 0 | 0 | 0 | 0 | 0 | 0 |
| 25, Effect of engagement with patients and others affected by the study | 0 | 0 | 0 | 0 | 0 | 0 | 0 | 0 | 0 |
| 26, Study findings, limitations, generalizability, and current knowledge | 1 | 1 | 1 | 1 | 1 | 1 | 0 | 1 | 1 |
| 27, Source of funding | 1 | 1 | 1 | 1 | 1 | 1 | 1 | 1 | 1 |
| 28, Conflicts of interest | 1 | 1 | 1 | 1 | 1 | 1 | 1 | 1 | 1 |
| **Total** | 10 | 9 | 9 | 10 | 10 | 9 | 8 | 10 | 9 |
| **% Reported (excl. N/A)** | 35.71% | 32.14% | 32.14% | 35.71% | 35.71% | 32.14% | 28.57% | 35.71% | 32.14% |

Abbreviations: N/A (Not Applicable); UTD (Unable to determine)

Notes:

From CHEERS (2022) checklist [43].

Not applicable (N/A) denotes when a checklist item is not relevant.
